# Morphology of the pancreas in type 2 diabetes: effect of weight loss with or without normalisation of insulin secretory capacity

**DOI:** 10.1007/s00125-016-3984-6

**Published:** 2016-05-14

**Authors:** Ahmad Al-Mrabeh, Kieren G. Hollingsworth, Sarah Steven, Roy Taylor

**Affiliations:** Magnetic Resonance Centre, Institute of Cellular Medicine, Campus for Ageing and Vitality, Newcastle University, Newcastle upon Tyne, NE4 5PL UK

**Keywords:** Fractal analysis, Magnetic resonance imaging, Pancreas morphology, Reversal of type 2 diabetes, Very low energy diet, Volume rendering

## Abstract

**Aims/hypothesis:**

This study was designed to establish whether the low volume and irregular border of the pancreas in type 2 diabetes would be normalised after reversal of diabetes.

**Methods:**

A total of 29 individuals with type 2 diabetes undertook a very low energy (very low calorie) diet for 8 weeks followed by weight maintenance for 6 months. Methods were established to quantify the pancreas volume and degree of irregularity of the pancreas border. Three-dimensional volume-rendering and fractal dimension (FD) analysis of the MRI-acquired images were employed, as was three-point Dixon imaging to quantify the fat content.

**Results:**

There was no change in pancreas volume 6 months after reversal of diabetes compared with baseline (52.0 ± 4.9 cm^3^ and 51.4 ± 4.5 cm^3^, respectively; *p* = 0.69), nor was any volumetric change observed in the non-responders. There was an inverse relationship between the volume and fat content of the pancreas in the total study population (r =−0.50, *p* = 0.006). Reversal of diabetes was associated with an increase in irregularity of the pancreas borders between baseline and 8 weeks (FD 1.143 ± 0.013 and 1.169 ± 0.006, respectively; *p* = 0.05), followed by a decrease at 6 months (1.130 ± 0.012, *p* = 0.006). On the other hand, no changes in FD were seen in the non-reversed group.

**Conclusions/interpretation:**

Restoration of normal insulin secretion did not increase the subnormal pancreas volume over 6 months in the study population. A significant change in irregularity of the pancreas borders occurred after acute weight loss only after reversal of diabetes. Pancreas morphology in type 2 diabetes may be prognostically important, and its relationship to change in beta cell function requires further study.

**Electronic supplementary material:**

The online version of this article (doi:10.1007/s00125-016-3984-6) contains peer-reviewed but unedited supplementary material, which is available to authorised users.

## Introduction

The pancreas remains one of the least studied organs in type 2 diabetes despite its central role in determining both onset and progression. This is largely a consequence of the difficulty of studying this organ in vivo due to its anatomical position surrounded by visceral fat deep in the abdominal cavity [1]. Only a small number of studies have employed MRI to quantify pancreas volume [2–5], including one study providing a validation of MRI quantification against direct water displacement measurement in mini-pigs [4]. We developed a robust MRI-based method for precise quantification of pancreas volume and observed a 33% decrease in type 2 diabetes compared with matched volunteers with normal glucose tolerance [6]. We also observed that the borders of the pancreas were markedly irregular compared with healthy individuals. The question must now be answered of whether these abnormalities are secondary to a decline in the trophic effects of mealtime spikes of intrapancreatic insulin concentration, or whether they may be primary. Given that insulin secretory function can now be returned to normal in type 2 diabetes with re-establishment of normal glucose control [7–9], it is now possible to address this question.

The original observation of irregular pancreas borders was made by simple visual inspection of images [6]. Although the etiolated appearance with serrated borders was striking, it was necessary to develop a quantitative method to allow precise description. Fractal dimension (FD) analysis has been used in biomedical imaging studies to address questions of biological relevance based on irregularity of borders with varied image complexity [10–12]. It has been successfully applied to histological study of the liver [13–16], pancreas [17] and spleen [18], and to CT- and MRI-generated images [11, 19–22]. The nature of the pancreas border introduces challenges to the application of FD analysis, and these have been overcome in this study.

The Counterbalance (Counteracting BetA cell failure by Long term Action to Normalize Calorie intakE) study observed the effect of a very low energy diet (very low calorie diet [VLCD]) on reversal of type 2 diabetes with follow-up over 6 months [9]. It demonstrated that those individuals achieving fasting plasma glucose <7 mmol/l after acute weight loss regained normal first-phase insulin secretion. MRI studies of the pancreas were carried out, allowing us to report for the first time on the effect on pancreas morphology of the duration of type 2 diabetes and of sustained restoration of insulin secretory function.

## Methods

### Study design and participants

The Counterbalance study was designed to examine the reversibility of type 2 diabetes in relation to disease duration, and the durability of reversal after following a VLCD. Full information on the study design and participants’ details has been published [8, 9]. In brief, 30 individuals (aged 35–70 years) with either short-term (0.5–4 years) or long-term (8–23 years) type 2 diabetes were studied (Table 1). MRI examination of the pancreas was performed at three time points: at baseline; following an 8 week VLCD and return to isoenergetic food intake; and following a structured, individualised weight maintenance programme over 6 months. All anthropometric measurements were made by a single observer (S. Steven). Thirty participants were initially studied, 29 of whom completed the study.

**Table 1:** Metabolic and anthropometric features of the studied population

Variable	Responders (*n* = 12)			Non-responders (*n* = 17)			Responders vs non-responders (*p* value)	
	Baseline	8 weeks	6 months	Baseline	8 weeks	6 months	Baseline	6 months
Weight (kg)	99.8 ± 3.2	84.1 ± 3.1*	84.4 ± 3.2*	96.7 ± 3.9	83.8 ± 3.4*	84.8 ± 3.7*	0.69	0.94
BMI (kg/m^2^)	34.0 ± 0.8	28.6 ± 0.8*	28.7 ± 0.7 *	34.4 ± 1.1	29.8 ± 1.1*	30.2 ± 1.1 *	0.75	0.33
Fasting plasma glucose (mmol/l)	8.9 ± 0.7	5.1 ± 0.2*	6.2 ± 0.3*	13.2 ± 0.6	8.5 ± 0.8*	9.4 ± 0.7*	0.004	0.01
Fasting insulin (pmol/l)	143.8 ± 23.6	63.2 ± 7.6*	68.8 ± 16.0*	78.5 ± 18.1	47.9 ± 8.3	49.3 ± 7.0	0.005	0.32
Total body fat (%)	36.2 ± 1.9	30.1 ± 2.0*	31.5 ± 1.9*	42.6 ± 2.2	37.2 ± 2.0*	40.8 ± 2.5	0.04	0.10
Pancreas fat (%)	4.5 ± 0.3	4.0 ± 0.3*	3.7 ± 0.3*	5.5 ± 0.8	5.5 ± 0.7	4.9 ± 0.6	0.97	0.27
HbA_1c_ (mmol/mol)	54.5 ± 3.7	39.8 ± 2.2*	41.4 ± 1.6*	68.1 ± 3.2	62.8 ± 4.3	61.7 ± 3.3	0.23	0.003
HbA_1c_ (%)	7.1 ± 0.3	5.8 ± 0.2	5.9 ± 0.2	8.4 ± 0.3	7.9 ± 0.4	7.8 ± 0.3	–	–
Triacylglycerol (mmol/l)	2.0 ± 0.3	1.0 ± 0.1*	1.2 ± 0.1 *	1.8 ± 0.4	1.0 ± 0.1*	1.2 ± 0.2*	0.83	0.55
Age (years)	52.0 ± 2.9	–	–	59.9 ± 2.1	–	–	0.03	–
Diabetes duration (years)	3.8 ± 1.0	–	–	9.8 ± 1.6	–	–	0.007	–
Sex (M/F)	8 M:4 F	–	–	7 M:10 F	–	–	–	–

Data are presented as mean ± SEM

The Student’s unpaired *t* test was used to calculate *p* values for difference in age and duration of diabetes, the Mann–Whitney *U* test was used to calculate *p* values for fasting insulin, and the Student’s paired *t* test was used for calculating the other *p* values

*p < 0.05 vs baseline

Participants were classified as either responders or non-responders based on a fasting plasma glucose level <7 mmol/l following a VLCD and return to normal diet [8, 9]. Responders were characterised by a shorter duration of diabetes (Table 1). The study protocol was approved by Newcastle and North Tyneside 2 Ethics Committee (REC 12/NE/0208), and all participants gave informed written consent before start of the study.

### MRI studies

MRI was performed using a 3.0 Tesla Philips Achieva scanner with a six-channel cardiac array for signal detection (Philips, Best, the Netherlands). Balanced turbo field echo structural axial images of the pancreas were generated as previously described, collecting 18 image sections of the abdomen [6]. MRI data were also acquired using a three-point Dixon method for fat quantification within the pancreas, as previously described [7].

### Pancreas volume quantification

Pancreas boundaries were manually delineated on each slice by selecting region of interest using the polygon tool of the open source ImageJ image analysis software [23], version 1.5b (http://rsb.info.nih.gov/ij/download.html). The total volume was obtained by summation of the delineated volumes on each slice after multiplication by the slice thickness, as previously reported [6]. All volumetric analyses were performed blind to the duration of diabetes or reversal status.

### Fractal analysis

The freely available Drishti Volume Exploration and Presentation Tool [24] was used for surface rendering of the pancreas image; version 2.5.1 (https://github.com/AjayLimaye/drishti/releases/tag/v2.5.1win) was used for analysis.The same software settings were used throughout the analysis of the whole study. A two-dimensional projection of the total extracted slices was generated in Drishti to represent the pancreas outline, and this was used for the FD analysis. This permitted mathematical expression of the irregularity of the three-dimensional organ in one position. FD analysis was carried using the FracLac plugin developed for ImageJ [25]; the July 2015 version was accessed (https://imagej.nih.gov/ij/plugins/fraclac/fraclac.html) and used throughout the study. The process is fully described in the electronic supplementary material (ESM) Methods and illustrated in ESM Figs 1 and 2. All image-processing steps and FD analyses were performed blind to the participant and the status of their diabetes. The previously reported visual semi-quantitative scoring scheme [6] was also applied to assess the irregularity of the pancreas borders, and the mean scores of two independent observers at baseline and at 6 months of study were compared with FD data.

### Fat quantification

Pancreas fat was quantified based on the three-point Dixon method as previously described [7].Two representative slices of pancreas were selected for fat quantification, and an improved method to select regions of homogenous intrapancreatic tissue was used. The oval tool of ImageJ was used to select three regions of interest (100 mm^2^ each) of uniform tissue from different locations of the pancreas to represent the whole organ. The average triacylglycerol content of three regions of interest within each slice was calculated, and the average of the two slices was used to derive the percentage pancreas triacylglycerol for each participant [26]. Total body fat mass was derived from the body composition using a Bodystat1500 (Bodystat, Douglas, Isle of Man, UK). Visceral fat percentage was measured using three-point Dixon imaging on representative slices at the L2–L3 vertebral level. The ImageJ polygon tool was used to select the region around the subcutaneous abdominal fat, selecting the region between the inner and outer subcutaneous fat boundaries to calculate subcutaneous fat and determine the percentage of visceral fat. All fat quantifications were performed blind to the duration of diabetes or reversal status.

### Analytical procedures

Plasma glucose concentrations were measured with a Yellow Springs glucose analyser (YSI, Ohio, USA). Other metabolic analyses were performed at a clinical pathology accredited laboratory (Newcastle upon Tyne Hospital NHS Foundation Trust, Department of Clinical Biochemistry).

### Statistical analysis

All data are presented as mean ± SEM. Statistical analysis was performed in Minitab version 16 (www.minitab.com). A *p* value <0.05 was considered statistically significant; a Student’s paired *t* test or Mann–Whitney U test was used to calculate the *p* value unless otherwise stated. Pearson’s or Spearman’s correlation coefficients were used as appropriate.

## Results

### Pancreas volume

**Fig. 1:**
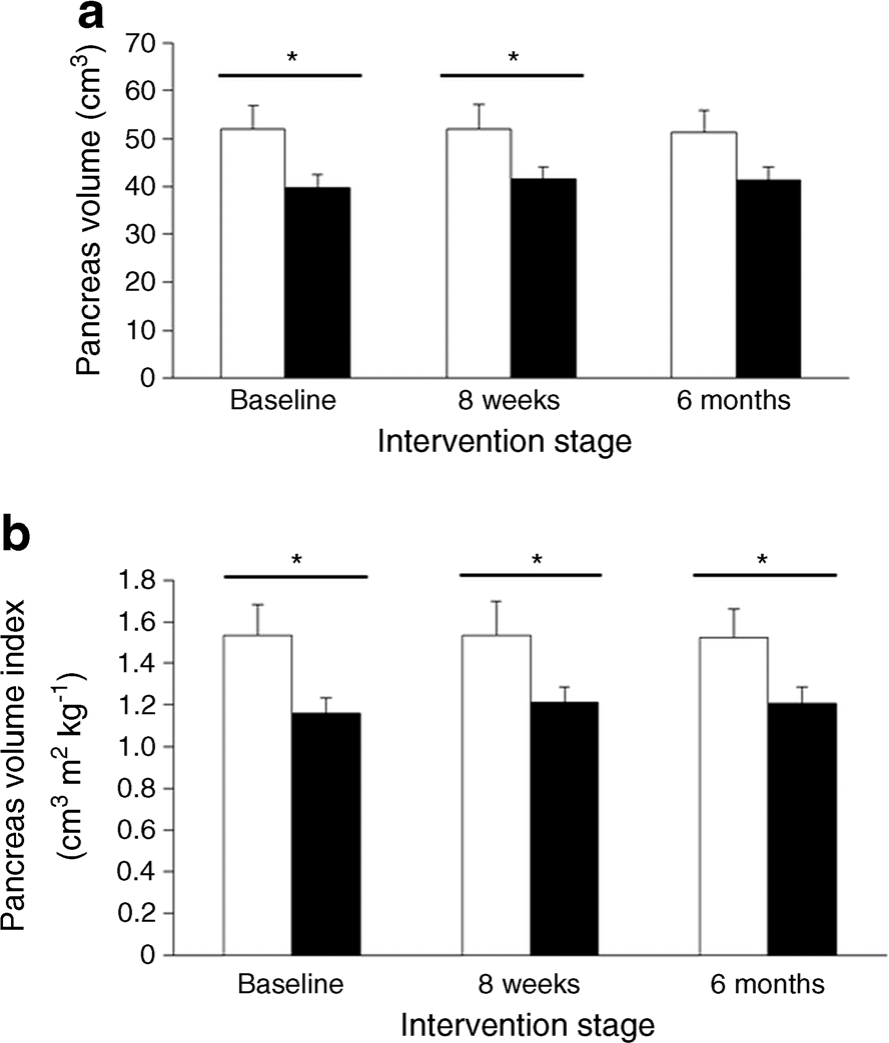
Difference in pancreas volume between the responder and non-responder groups at different stages of the study. (**a**) Absolute pancreas volume change between responders (white bars) and non-responders (black bars) at baseline, 8 weeks and 6 months. (**b**) Change in pancreas index (pancreas volume/BMI at baseline) between responders and non-responders at baseline, 8 weeks and 6 months. Error bars represent SEMs. *p < 0.05 as shown

At baseline, the mean pancreas volume was greater in the responders than the non-responders (52.0 ± 4.9 cm^3^ vs 39.7 ± 2.7 cm^3^; *p* < 0.05; Fig. 1a). The relative difference was similar when normalised using BMI to give the pancreas volume index (1.54 ± 0.15 vs 1.16 ± 0.16 cm^3^ m^2^ kg^−1^; *p* < 0.02; Fig. 1b). After the VLCD, the pancreas volume was unchanged in both the responders (52.0 ± 4.9 cm^3^, 51.9 ± 5.3 cm^3^, 51.4 ± 4.5 cm^3^) and non-responders (39.7 ± 2.7 cm^3^, 41.4 ± 2.6 cm^3^, 41.3 ± 2.8 cm^3^; Fig. 1a) at baseline, 8 weeks, and 6 months, respectively.

### Pancreas volume and clinical characteristics

Pancreas volume at baseline was inversely related to duration of diabetes (r =−0.46, *p* = 0.03) and tended to increase with BMI. There was no significant correlation between pancreas volume and age (r =−0.16, *p* = 0.46) and no significant difference in pancreas volume between men and women (47.4 ± 3.9 vs 41.1 ± 3.8 cm^3^ ; *p* = 0.27). There was a weak correlation at baseline between fasting insulin level and pancreas volume in the total study population (r = 0.35, *p* = 0.06) but no correlation within each group (responders, r =−0.007, *p* = 0.98; non-responders, r = 0.30, *p* = 0.24). There was no relationship between pancreas volume and fasting plasma glucose level for the total study population or for the separate groups.

### Relationship between pancreas volume and fat content

**Fig. 2:**
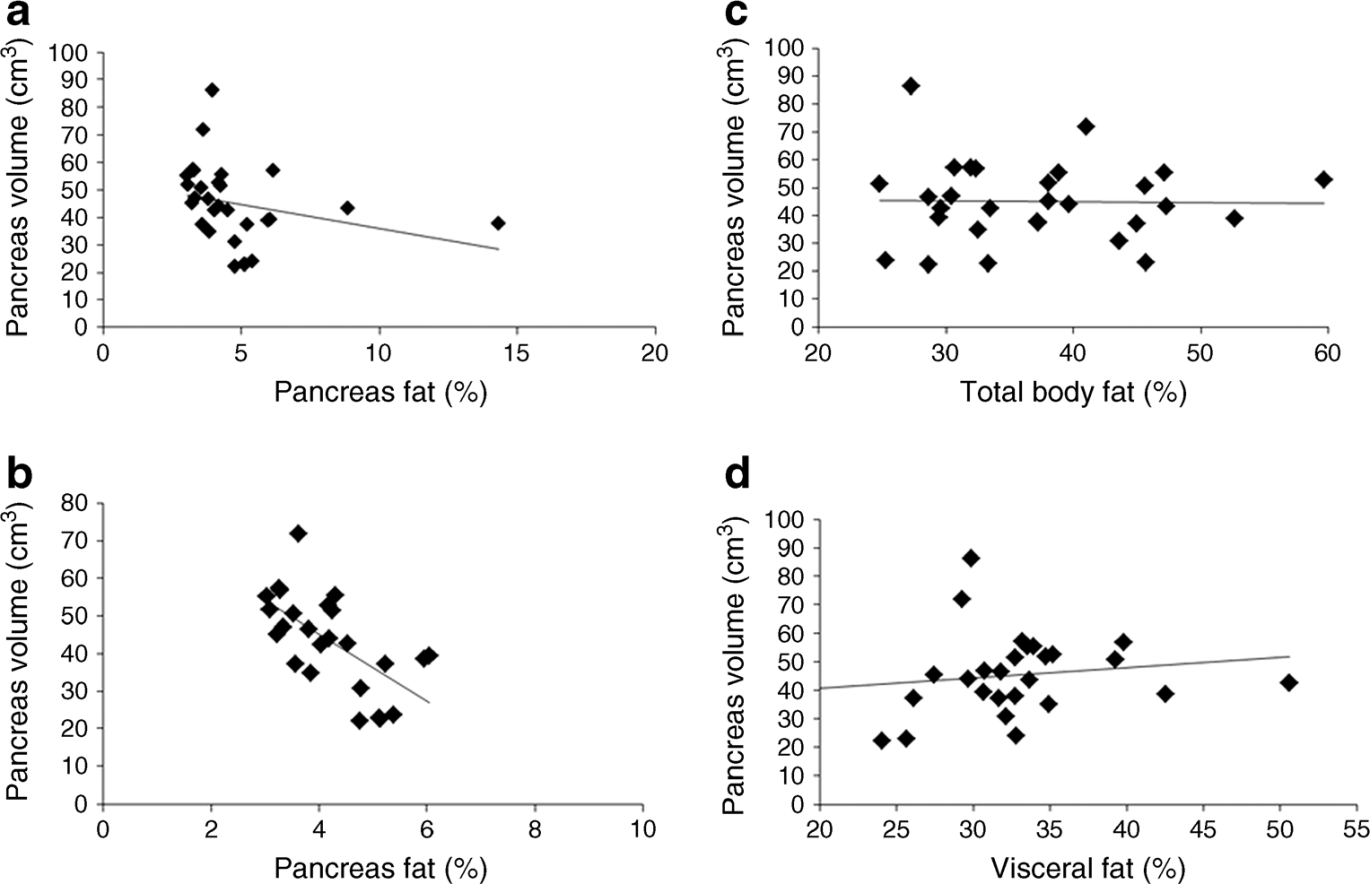
Relationship between pancreas volume and fat content. (**a**, **b**) Relationship between pancreas volume (cm^3^) and pancreas fat (%) in the total study population and after removing the outliers, respectively. (**c**) Relationship between pancreas volume (cm^3^) and percentage of total body fat in the total study population. (**d**) Relationship between pancreas volume (cm^3^) and percentage of visceral fat in the total study population. The average of fat (%) and pancreas volume at all stages is presented. Correlation coefficients are: (**a**) *r* = −0.5, *p* = 0.006; (**b**) *r* = −0.67, *p* < 0.0001; (**c**) *r* = −0.01, *p* = 0.96; (**d**) *r* = 0.18, *p* = 0.36

The pancreas volume was inversely proportional to the pancreas fat content (r =−0.50, *p* = 0.006; Fig. 2a). Two individuals had high pancreas fat levels, but the correlation did not depend on these as excluding the outliers strengthened the correlation (r =−0.7, *p* < 0.0001; Fig. 2b). No significant correlation was observed between pancreas volume and either total body or visceral fat (Fig. 2c, d).

### Change in pancreas borders

**Fig. 3:**
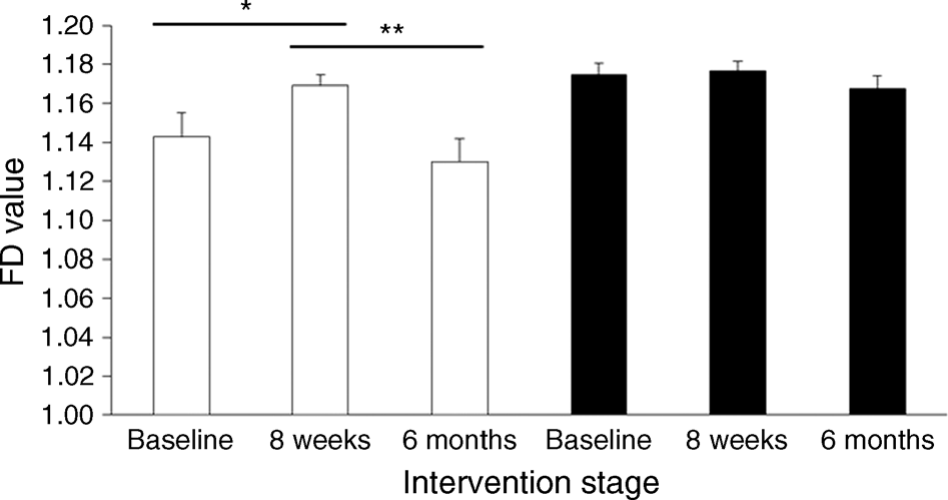
Changes in FD between responders and non-responders at different stages of the study. FD was measured on two images of the pancreas for every participant, and the average of the two results is presented. Mean values are presented, and error bars represent SEMs. Responders (white bars); non-responders (black bars). *p < 0.05, **p < 0.01 as shown

The CV for the FD analysis of two replicate images at the study baseline was 0.88%, demonstrating high precision of the FD analysis method. In the responders, mean FD increased from 1.143 ± 0.013 at baseline to 1.169 ± 0.006 at 8 weeks, after the VLCD (*p* = 0.05), indicating an increase in irregularity of the pancreas margins. This was followed by a greater decrease in FD between 8 weeks and 6 months (to 1.130 ± 0.012, *p* = 0.006; Fig. 3, white bars). In the non-responders, mean FD did not change (1.175 ± 0.006, 1.176 ± 0.005 and 1.167 ± 0.007 at baseline, 8 weeks and 6 months; Fig. 3, black bars). At all time points, the extent of the pancreas border abnormality was higher in the non-responders than the responders (Fig. 3), and the difference increased during the study (*p* = 0.01 at 6 months). The decrease in FD in responders was associated with a decrease in fasting plasma insulin level between baseline and 6 months (143.8 ± 23.6 to 68.8 ± 16.0 pmol/l; *p* = 0.007; Table 1), while the insulin level did not significantly change in non-responders (from 78.5 ± 18.1 to 49.3 ± 7.0 pmol/l; *p* = 0.28).

**Fig. 4:**
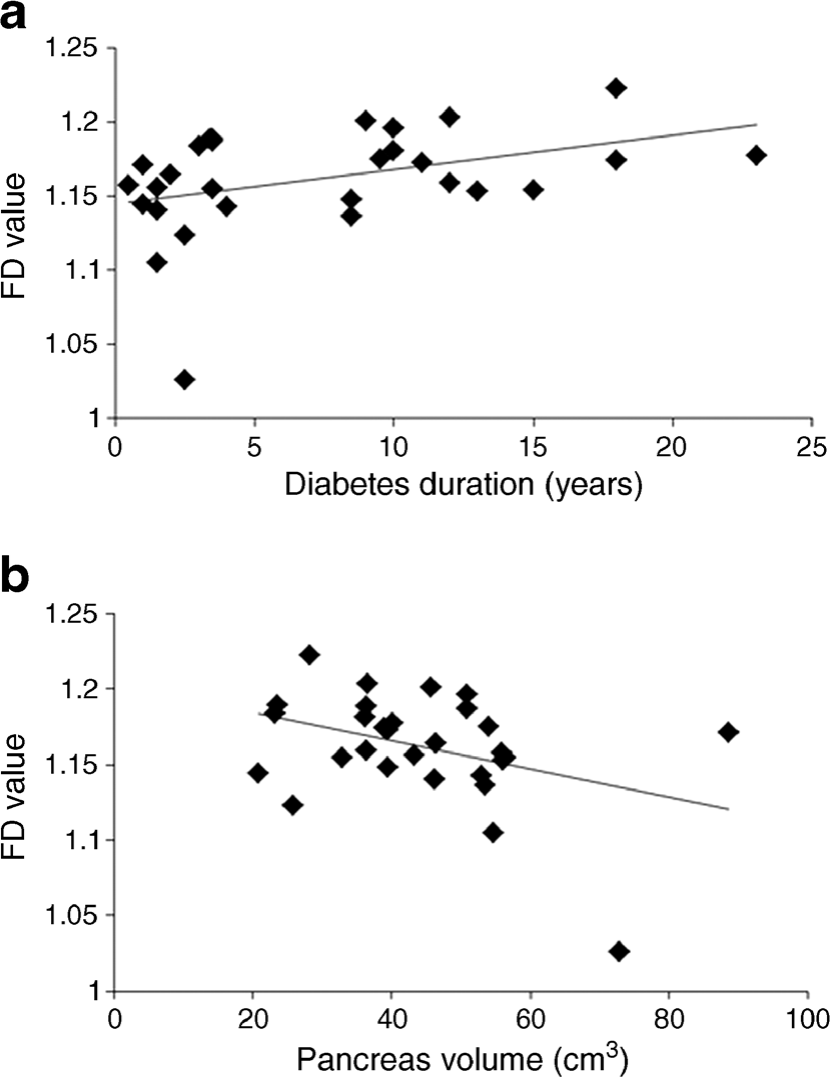
Relationship between FD, pancreas volume (cm^3^) and duration of diabetes. FD was measured on two images of the pancreas for every participant, and the average of the two results is presented. All data are presented at the study baseline. Correlations between (**a**) FD and duration of diabetes: *r* = 0.42, *p* = 0.02; (**b**) FD and pancreas volume: *r* = −0.38, *p* = 0.04. Excluding the outlier with very large pancreas volume from (**b**) enhanced the correlation (*r* = −0.50, *p* = 0.007)

At baseline, FD correlated positively with duration of diabetes (r = 0.42, *p* = 0.02; Fig. 4a). In addition, there was an inverse relationship between FD and pancreas volume (r =−0.38, *p* < 0.05; Fig. 4b).

The semi-quantitative visual scoring scheme showed agreement with the FD data, the mean score in responders being 2.2 at baseline and increasing to 2.9 by the end of 6 months (*p* < 0.05). On the other hand, no significant change was observed in the non-responders (mean scores 2.1 vs 2.3 respectively; *p* > 0.05).

## Discussion

This study has established that pancreas volume is a marker of reversibility of type 2 diabetes, being higher in responders. However, in those individuals who regain normal insulin secretion after weight loss, there is no detectable increase in pancreas volume over 6 months. The irregularity of the pancreas border has been quantified and shown to change over 6 months in individuals who show a reversal of their diabetes, but to remain unchanged despite a similar weight loss in those who remain diabetic after their weight loss.

In type 2 diabetes, studies using ultrasound, CT and MRI have suggested a 7–22% decrease in pancreas volume [5, 27–30]. Previously, we observed a 33% decrease in pancreas volume in individuals who had type 2 diabetes compared with matched controls with normal glucose tolerance [6]. In the present study, which included patients with a longer duration of type 2 diabetes, we observed that the pancreas volume decreased by almost 50% relative to the previously reported non-diabetic control group (44.4 ± 2.8 vs 82.6 ± 4.9 cm^3^; *p* < 0.001). Reflecting this, a significant inverse correlation between pancreas volume and duration of diabetes was defined within the present study population. Although a large study of individuals up to the age of 100 years showed an age-related decline in pancreas volume [28], within the 35–70 year age range of the present study this did not explain the duration-related decrease. Despite the restoration of acute insulin responsiveness to plasma glucose concentration, pancreas volume based on manual delineation of the pancreas borders did not change.

One aim of the present study was to determine whether the decrease in volume of the pancreas was a consequence of the ongoing pathological processes or whether individuals with a small pancreas might be predisposed to developing type 2 diabetes. Pancreas volume could secondarily be affected by a loss of the normal postprandial rise in insulin, acting by a paracrine effect. Insulin is a potent stimulator of growth at concentrations around ten-fold greater than those required for metabolic effects [31]. Pancreas tissue is likely to be exposed to very high concentrations of insulin after meals, when the local rise in concentration must exceed the 10–15-fold rise achieved in plasma concentration by at least two orders of magnitude [32]. When there is no local insulin production in type 1 diabetes, pancreas volume is known to be decreased by one third [33] and this is evident from just after diagnosis [3]. In type 2 diabetes, basal insulin levels are raised, but there is an absence of the immediate and major insulin release after meals that could confer local growth-promoting effects of the hormone. There are few animal studies of pancreas volume, although a study in non-human primates observed a modest decrease in pancreas volume 2–6 months after inhibition of insulin secretion by low-dose streptozotocin [34]. The hypothesis that the restoration of large meal-related increases in intra-pancreatic insulin concentration might restore the trophic effects of insulin and hence normal pancreas volume has been disproved by the present study, at least over a 6 month period. As pancreas volume decreased with increasing duration of diabetes, it appears likely that loss of volume is indeed secondary to the disease rather than being a factor increasing susceptibility to developing the condition. However, future studies must define pancreas volume in populations at risk of developing type 2 diabetes.

There is a marked variation of pancreatic morphology in the general population, with a more serrated boundary of the pancreas generally ascribed to ageing [35]. Anecdotally, radiologists recognise that the serrated appearance is also associated with diabetes. We previously reported a significantly greater irregularity of the pancreas border in type 2 diabetes using a semi-quantitative visual inspection method [6]. To quantify the irregularity in an objective manner, we employed FD analysis based on a standard box counting method [19–22]. By a combination of three-dimensional volume segmentation of the pancreas and FD analysis, we developed a new platform that can mathematically define the morphology of the pancreas borders. Unexpectedly, FD analysis showed a significant increase in pancreas complexity after acute weight loss only in the responder group. This was followed by a significant decrease, such that by the end of the study the pancreas border had become smoother. No change in the pancreas border was seen in the non-responder group. Further work is required to determine whether this pattern of change relates to an acute loss of intrapancreatic fat with underlying slow hypertrophy of pancreas tissue continuing over 6 months.

There was a negative correlation between FD value and pancreas volume in the total study population at baseline, while FD increased in proportion to duration of diabetes. The latter can explain the significant difference in FD value between the groups with short-term and long-term diabetes. Regeneration of pancreatic tissues has been reported to affect both the endocrine and exocrine elements [36]. The present observation that FD change occurred only in the responder group excludes the possibility that change in FD is solely the result of a loss of visceral fat. Longer term follow-up after reversal of type 2 diabetes will be required to determine the extent and prognostic significance of a decrease in irregularity of the pancreas border.

The limitations of this study need to be highlighted. First, although we have previously demonstrated good precision of the method in quantifying pancreas volume [6], accuracy is more difficult to establish in humans. Volumetry by MRI has been shown to correspond almost exactly to that by water displacement for the pancreas of mini-pigs [4]. Second, it is likely that the volume of the pancreas had declined very slowly over a decade or more, and follow-up observation for longer than 6 months is now required. Third, it is conceivable that a true change in pancreas volume after reversal of type 2 diabetes may be below the precision power of this quantification method. Work is underway to further optimise image acquisition for the three-dimensional volume-rendering method. The latter technique requires less human input and could potentially generate more accurate volumetric data than the contour-based ImageJ method. Fourth, FD analysis has not previously been applied to study of the pancreas. In the current study, we measured FD on only part of the pancreas surface exposed as the edge of the pancreas on each projection. Further work is required to optimise the method by applying fractal analysis to the whole three-dimensional rendered pancreas to measure the global FD change.

In conclusion, this study quantifies the extent of a decrease in pancreas volume and irregularity of the pancreas borders in people with type 2 diabetes defined in terms of the capacity to regain beta cell function following weight loss. Increasing duration of diabetes was associated with both lower pancreas volume and greater irregularity of the pancreas border. Reversal of type 2 diabetes over a 6 month period brought about smoothing of the pancreas border only in individuals who achieved a return to normal metabolic regulation. No detectable increase in pancreas volume occurred after weight loss irrespective of the metabolic improvement.

## Electronic supplementary material

Below is the link to the electronic supplementary material.

ESM Methods (PDF 84.1 kb)

ESM Fig. 1 (PDF 343 kb)

ESM Fig. 2 (PDF 390 kb)

## Abbreviations

FD: Fractal dimension
VLCD: Very low calorie diet

## References

[1] Hruban RH (2004). History of the pancreas: mysteries of a hidden organ. Bull Hist Med.

[2] Williams AJ, Chau W, Callaway MP, Dayan CM (2007). Magnetic resonance imaging: a reliable method for measuring pancreatic volume in type 1 diabetes. Diabet Med.

[3] Williams AJ, Thrower SL, Sequeiros IM (2012). Pancreatic volume is reduced in adult patients with recently diagnosed type 1 diabetes. J Clin Endocrinol Metab.

[4] Szczepaniak EW, Malliaras K, Nelson MD, Szczepaniak LS (2013). Measurement of pancreatic volume by abdominal MRI: a validation study. PLoS One.

[5] Burute N, Nisenbaum R, Jenkins DJ (2014). Pancreas volume measurement in patients with type 2 diabetes using magnetic resonance imaging-based planimetry. Pancreatology.

[6] Macauley M, Percival K, Thelwall PE, Hollingsworth KG, Taylor R (2015). Altered volume, morphology and composition of the pancreas in type 2 diabetes. PLoS One.

[7] Lim EL, Hollingsworth KG, Aribisala BS, Chen MJ, Mathers JC, Taylor R (2011). Reversal of type 2 diabetes: normalisation of beta cell function in association with decreased pancreas and liver triacylglycerol. Diabetologia.

[8] Steven S, Taylor R (2015). Restoring normoglycaemia by use of a very low calorie diet in long- and short-duration type 2 diabetes. Diabet Med.

[9] Steven S, Hollingsworth KG, Al-Mrabeh A (2016). Very low calorie diet and 6 months of weight stability in type 2 diabetes: pathophysiologic changes in responders and non-responders. Diabetes Care.

[10] Lopes R, Betrouni N (2009). Fractal and multifractal analysis: a review. Med Image Anal.

[11] Lennon FE, Cianci GC, Cipriani NA (2015). Lung cancer—a fractal viewpoint. Nat Rev Clin Oncol.

[12] Weibel E, Losa G, Merlini D, Nonnenmacher T, Weibel E (2005). Mandelbrot’s fractals and the geometry of life: a tribute to Benoît Mandelbrot on his 80th birthday. Fractals in biology and medicine.

[13] Dioguardi N, Franceschini B, Aletti G, Russo C, Grizzi F (2003). Fractal dimension rectified meter for quantification of liver fibrosis and other irregular microscopic objects. Anal Quant Cytol Histol.

[14] Dioguardi N, Grizzi F, Franceschini B, Bossi P, Russo C (2006). Liver fibrosis and tissue architectural change measurement using fractal-rectified metrics and Hurst’s exponent. World J Gastroenterol.

[15] Soda G, Nardoni S, Bosco D, Grizzi F, Dioguardi N, Melis M (2003). Fractal analysis of liver fibrosis. Pathologica.

[16] Moal F, Chappard D, Wang J (2002). Fractal dimension can distinguish models and pharmacologic changes in liver fibrosis in rats. Hepatology.

[17] Pantic I, Nesic D, Stevanovic D, Starcevic V, Pantic S, Trajkovic V (2013). Effects of ghrelin on the structural complexity of exocrine pancreas tissue architecture. Microsc Microanal.

[18] Pantic I, Paunovic J, Basta-Jovanovic G, Perovic M, Pantic S, Milosevic NT (2013). Age-related reduction of structural complexity in spleen hematopoietic tissue architecture in mice. Exp Gerontol.

[19] Davnall F, Yip CS, Ljungqvist G (2012). Assessment of tumor heterogeneity: an emerging imaging tool for clinical practice?. Insights Imaging.

[20] Captur G, Muthurangu V, Cook C (2013). Quantification of left ventricular trabeculae using fractal analysis. J Cardiovasc Magn Reson.

[21] Rajagopalan V, Liu Z, Allexandre D (2013). Brain white matter shape changes in amyotrophic lateral sclerosis (ALS): a fractal dimension study. PLoS One.

[22] Al-Kadi OS, Watson D (2008). Texture analysis of aggressive and nonaggressive lung tumor CE CT images. IEEE Trans Biomed Eng.

[23] Schneider CA, Rasband WS, Eliceiri KW (2012). NIH Image to ImageJ: 25 years of image analysis. Nat Methods.

[24] Limaye A (2012) Drishti: a volume exploration and presentation tool. Proc. SPIE 8506, Developments in X-Ray Tomography VIII, 85060X (October 17, 2012); doi:10.1117/12.935640

[25] Karperien A (2013) Fractal dimension and lacunarity. Available from http://rsbweb.nih.gov/ij/plugins/fraclac/fraclac.html, accessed 10 September 2015

[26] Hollingsworth KG, Al-Mrabeh A, Steven S, Taylor R (2015). Pancreatic triacylglycerol distribution in type 2 diabetes. Diabetologia.

[27] Alzaid A, Aideyan O, Nawaz S (1993). The size of the pancreas in diabetes mellitus. Diabet Med.

[28] Saisho Y, Butler AE, Meier JJ (2007). Pancreas volumes in humans from birth to age one hundred taking into account sex, obesity, and presence of type-2 diabetes. Clin Anat.

[29] Goda K, Sasaki E, Nagata K, Fukai M, Ohsawa N, Hahafusa T (2001). Pancreatic volume in type 1 and type 2 diabetes mellitus. Acta Diabetol.

[30] Lim S, Bae JH, Chun EJ (2014). Differences in pancreatic volume, fat content, and fat density measured by multidetector-row computed tomography according to the duration of diabetes. Acta Diabetol.

[31] Poggi C, Le Marchand-Brustel Y, Zapf J, Froesch ER, Freychet P (1979). Effects and binding of insulin-like growth factor I in the isolated soleus muscle of lean and obese mice: comparison with insulin. Endocrinology.

[32] Carey PE, Gerrard J, Cline GW (2005). Acute inhibition of lipolysis does not affect postprandial suppression of endogenous glucose production. Am J Physiol Endocrinol Metab.

[33] Foulis AK, Frier BM (1984). Pancreatic endocrine-exocrine function in diabetes: an old alliance disturbed. Diabet Med.

[34] Saisho Y, Manesso E, Butler AE (2011). Ongoing beta-cell turnover in adult nonhuman primates is not adaptively increased in streptozotocin-induced diabetes. Diabetes.

[35] Hahn PF, Stark DD, Bradley WG (1999). Biliary system, pancreas, spleen and alimentary tract. Magnetic resonance imaging.

[36] Murtaugh LC, Keefe MD (2015). Regeneration and repair of the exocrine pancreas. Annu Rev Physiol.

