# Supplementary material for: Morphology of the pancreas in type 2 diabetes: effect of weight loss with or without normalisation of insulin secretory capacity
Source: Diabetologia. 2016 May 14;59:1753–9. doi: 10.1007/s00125-016-3984-6 (PMC4930466; doi:10.1007/s00125-016-3984-6)
Supplement: Supplementary file 1 — (PDF 84.1 kb) [file 125_2016_3984_MOESM1_ESM.pdf]

Methods

Fractal dimension (FD) analysis was performed using a standard box counting method on two-dimensional projection image of the volume rendered pancreas. BTFE axial image (ESM Fig. 1a) was processed first in Drishti to extract volume of the total pancreas slices then two-dimensional projection images were created from the extracted volume (ESM Fig. 1b). The generated image was first processed in Matlab (MathWorks, Cambridge, UK) using an algorithm [18] which generates a grayscale image which highlights the borders of the pancreas (ESM Fig. 1c).The math tool of ImageJ was then used to eliminate features from the interior part of the image leaving only the highlighted borders (ESM Fig.1d). A standard box-counting method was employed to measure FD of the pancreas borders using the ImageJ developed FracLac plugin [21]. FD analysis was performed by an automated method using the batch processing option, and by following the basic operating procedures. Image was first binarized automatically by selecting auto convert to binary option (ESM Fig.1e), other default software settings were applied including applying 12 grids per image, box size (scale) varied between 0-45% of the image size, and rotation at 36° angle to generate 10 rotated grids for each image (ESM Fig.1f), average FD values were calculated for the 12 grids per rotation (120 grids per scale) and the logarithmic relationship between average box count and box size was computed. The line of best fit of box count/box size relationship was drawn and the FD is calculated as the slope of this line (ESM Fig.1g). FD was performed on two differently oriented images, and average FD of both images was presented as the final FD value, a more detailed illustration of how FD was performed is shown in ESM Figure 1 and 2. All image processing steps and FD analysis were performed blinded to participant and diabetes status.
