# Supplementary material for: Morphology of the pancreas in type 2 diabetes: effect of weight loss with or without normalisation of insulin secretory capacity
Source: Diabetologia. 2016 May 14;59:1753–9. doi: 10.1007/s00125-016-3984-6 (PMC4930466; doi:10.1007/s00125-016-3984-6)
Supplement: Supplementary file 2 — (PDF 343 kb) [file 125_2016_3984_MOESM2_ESM.pdf]

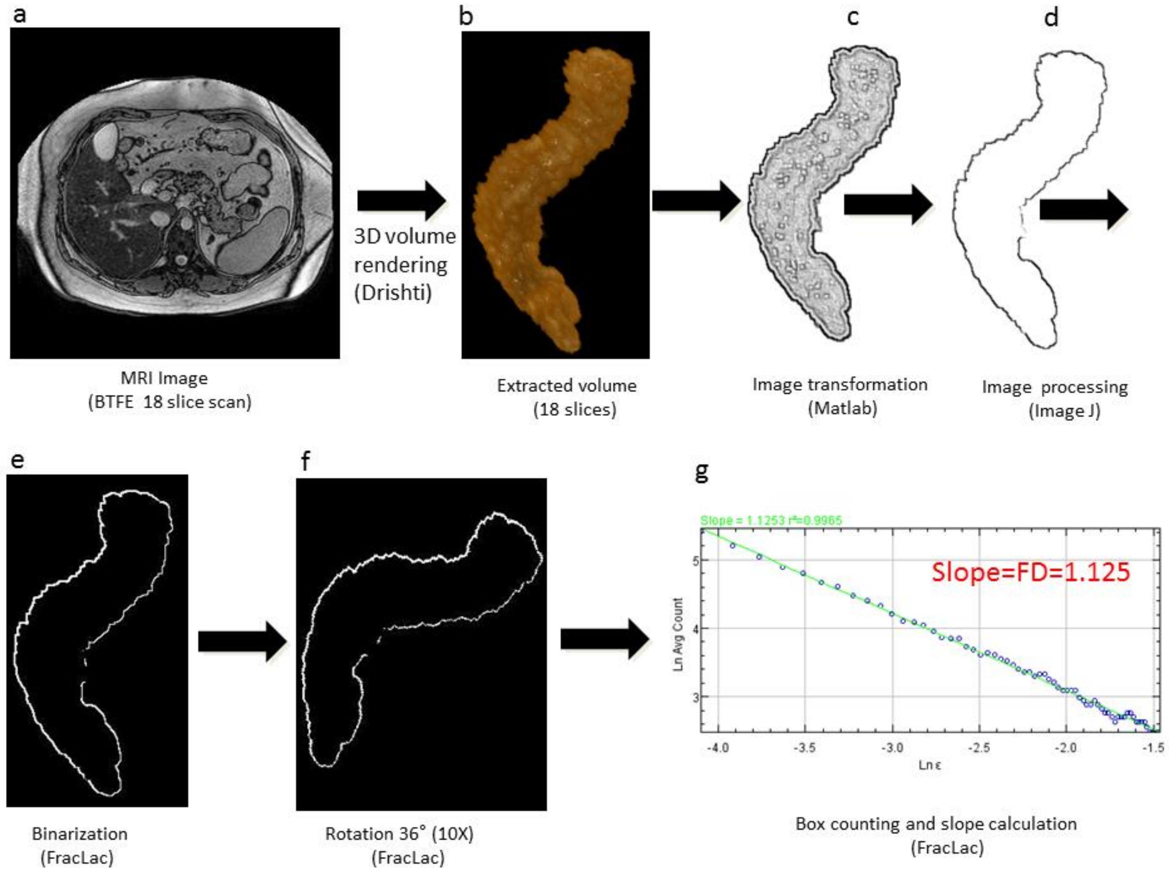

ESM Figure 1: Steps in image processing in preparation for fractal analysis of the pancreas borders

(a) 18 BTFE axial (5mm thick) slices of the abdomen MRI image covering the whole pancreas from the tail to the head of type 2 diabetes patient. (b) Two-dimensional projection image generated from the three-dimensional volume rendering of total pancreas slices using Drishti volume exploration and presentation tool. (c) The Two-dimensional projection image was processed in Matlab to generate grayscale image with clear borders. (d) Image was further processed in ImageJ to eliminate unwanted internal features and keep the borders only. (e) Image was automatically binarized by FracLac. (f) Image was automatically rotated (10x) at 36 ^{o} angle in FracLac. (g) FD calculation of the average rotation in FracLac (ε= box size, avg count= number of boxes per scale).
