# Supplementary material for: Morphology of the pancreas in type 2 diabetes: effect of weight loss with or without normalisation of insulin secretory capacity
Source: Diabetologia. 2016 May 14;59:1753–9. doi: 10.1007/s00125-016-3984-6 (PMC4930466; doi:10.1007/s00125-016-3984-6)
Supplement: Supplementary file 3 — (PDF 390 kb) [file 125_2016_3984_MOESM3_ESM.pdf]

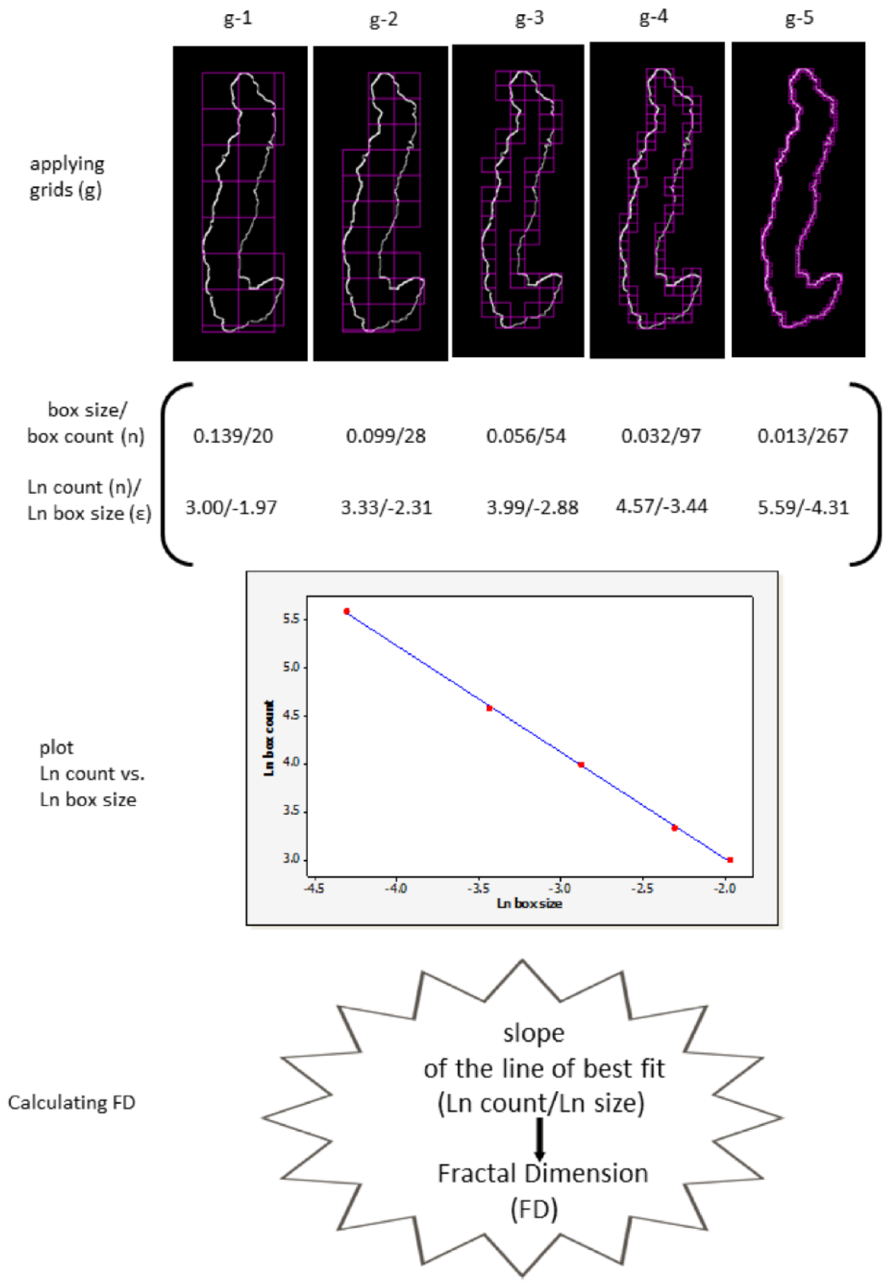

ESM Figure 2: Illustration of the basic steps in measuring FD of the pancreas using standard box counting method

A grid of varied box size (scale) between 0- 45% of the image size was displayed on the binarized image and the number of boxes (n) that contain non-zero pixels was counted for each grid (g). The Ln of box count (n) was plotted against the Ln of box size (ε), and the slope of the regression line was presented as the final FD value. Only 5 iterations were presented in this illustrative example, but in reality the number of iterations is higher depending on the scale which is decided by the software based on the image size. In this example, grids were placed randomly in one position on the image. However, in practice each grid is placed at 12 different locations and rotated 10 times at 36° angle, and the average values for 10 rotations / 12 positions are calculated.
